# Genetic Variants of Glycogen Metabolism Genes Were Associated With Liver PDFF Without Increasing NAFLD Risk

**DOI:** 10.3389/fgene.2022.830445

**Published:** 2022-04-06

**Authors:** Liu Yang, Zewen Sun, Jiuling Li, Xingchen Pan, Jianping Wen, Jianli Yang, Qing Wang, Peng Chen

**Affiliations:** ^1^ Department of Endocrinology, China-Japan Union Hospital, Jilin University, Changchun, China; ^2^ Department of Genetics, College of Basic Medical Sciences, Jilin University, Changchun, China; ^3^ Key Laboratory of Pathobiology, Ministry of Education, College of Basic Medical Sciences, Jilin University, Changchun, China; ^4^ Department of Pathology, College of Basic Medical Sciences, Jilin University, Changchun, China; ^5^ Department of Molecular Biology, College of Basic Medical Sciences, Jilin University, Changchun, China

**Keywords:** non-alcoholic fatty liver disease, liver fat content, magnetic resonance imaging, proton density fat fraction, glycogen synthase, glycogen phosphorylase, glycogen metabolism

## Abstract

**Background/Aims:** The storage amount of liver glycogen could affect the liver fibrosis assessment made by MRI-based methods. However, it remained unclear whether glycogen amount could bias the estimation of liver fat content by proton density fat fraction. In this study, we aimed to investigate whether glycogen metabolism gene variants could contribute to the bias of PDFF by genetic association.

**Methods:** We conducted an association study of the glycogen metabolism genes based on the PDFF data of 11,129 participants in the UK Biobank. The effect of the SNPs in these genes on non-alcoholic fatty liver disease was estimated by a meta-analysis of the available NAFLD case-control studies.

**Results:** We identified significant associations of the SNPs near the genes encoding glycogen phosphorylase (*PYGM* and *PYGL*) and synthase (*GYS2*) with PDFF (FDR-corrected *p* value < 0.05). The genes encoding the regulatory proteins of glycogenolysis (*PHKB*, *CALM2/3*), glucose transporter (*SLC2A1*), and glucose kinase (*GCK*) were also associated with PDFF. The SNP rs5402 of *SLC2A2* and rs547066 of *PYGM* were associated with NAFLD (*p* < 0.05) with others being insignificant. Except for the *PYGM* gene, the PDFF-associated SNPs showed no associations with NAFLD. In addition, the burden tests of rare variants in these genes were not significant after FDR correction.

**Conclusion:** Liver glycogen metabolism genes associated with PDFF were not associated with NAFLD, which implicated a potential bias effect of glycogen storage on the quantification of liver fat content by PDFF.

## Introduction

Non-alcoholic fatty liver disease (NAFLD) is the condition in which liver fat content exceeds 5% of liver weight in the absence of excessive alcohol consumption, viral infection, or chemical or physical liver damages (Mundi et al., 2020). Currently, liver biopsy is the gold standard for the diagnosis of NAFLD. Nonetheless, the need for noninvasive methods to identify liver steatosis has been emerging to overcome the shortcomings of liver biopsy, including bleeding and complications, inter-observation variability.

Magnetic resonance imaging proton density fat fraction (MRI-PDFF) is a quantitative imaging biomarker and is an accurate and highly sensitive method for measuring the steatosis of the whole liver. It can accurately detect microscopic steatosis as small as 5% (Schwimmer et al., 2005; Le et al., 2012; Reeder et al., 2012; Reeder 2013). MRI-PDFF is increasingly used in the diagnosis of fatty liver diseases and in clinical trials to evaluate the drug efficacy. Previous studies have shown that hepatic glycogen content could affect T1 MRI mapping, which in theory could induce errors in PDFF measurements (Kramer et al., 2017; Mozes et al., 2021). At present, direct quantification of liver glycogen is possible through PAS staining or biochemical measurements of liver biopsy, or ^13^C-MRS *in vivo*. However, the evidence for the impact of hepatic glycogen content on MRI-PDFF measurements has not been demonstrated.

Here, we investigated whether glycogen metabolism genes could potentially affect the liver fat content assessed by imaging-based methods using genetic association studies of PDFF and NAFLD. With this genetic evidence, our study will be informative for the rational of considering glycogen storage when developing novel imaging-based diagnostic methods or the clinical interpretation of current PDFF measurements.

## Materials and Methods

### The UK Biobank Cohort

The UK Biobank is a prospective cohort study that recruited more than 500,000 participants aged 37–73 (99.5% of people aged 40–69) from all over the United Kingdom from 2006 to 2010. In 2014, approximately 45,000 participants accepted the UK Biobank’s invitation for a visit of abdominal magnetic resonance imaging. Although the invitation was not determined by medical information, the MRI exclusion criteria (such as metal or electronic implants, surgery 6 weeks before the appointment, severe hearing, or respiratory dysfunction) may lead to a slightly healthy cohort. The UK Biobank has been approved by the Northwest Multicenter Research Ethics Committee (reference number: 11/NW/0382) and obtained written informed consent from all the participants before the study.

### Proton Density Fat Fraction Measurement

Dixon sequences were acquired using Siemens 1.5T MAGNETOM Aera at the dedicated UK Biobank imaging center (Wilman et al., 2017). PDFF from DIXON sequence were calculated using LiverMultiScan (Perspectum Diagnostics, Oxford, United Kingdom). The multi-gradient GRE was used to scan the transverse section of the liver center above the hilum using the extended 3D DIXON sequence with acquisition parameters of 2.5 mm voxel size, 6 mm slice thickness, 20° flip angle (FA), 27 ms repetition time (TR), and two signal averages. The second, fourth, and sixth echoes were used to construct the PDFF map. Three 15 mm diameter circular regions of interest (ROI) were selected by trained professionals to cover the liver parenchyma and avoid blood vessels or bile ducts in the PDFF map. The reported PDFF was calculated based on the average density of the three ROIs from the fat and water phases, using the liver fat percentage formula: fat/(water + fat). PDFF≥5% was defined as fatty liver disease. In total, we have the PDFF measurements for 16,307 individuals.

### Genetic Data and QC

The genetic data and quality control procedure have been described elsewhere (Bycroft et al., 2018). In brief, the genomic DNA was extracted from peripheral blood and genotyped by Applied Biosystems UK Biobank Axiom Array (*N* = 14,328) and UK BiLEVE Axiom Array (*N* = 1,517). Individuals whose genotype missing rate >5%), heterogeneity rate >3 SD, or self-reported sex mismatched the one inferred from the genotypes were excluded. The included participants were of European ancestry. The genotypes were imputed off the combined haplotype reference of the UK10K and 1000 genome cosmopolitan panels. Our SNP association analysis was restricted to the genetic variations with minor allele frequency (MAF) > 1% and imputation quality score> 0.7. The genetic principal components were calculated from the genotypes of the UK Biobank array data (Abraham and Inouye 2014)

### Candidate Gene and SNP Selection

Human genes encoding the enzymes for glycogenesis and glycogenolysis, as well as the direct regulator factors of these reversible reactions, were selected as the candidate genes according to the pathway map of Kyoto Encyclopedia of Genes and Genomes (https://www.kegg.jp). For the purpose of this study, we restricted the candidate genes in autosomes, which resulted in the removal of *PHKA1* and *PHKA2*. Both of these genes are located in chromosome X. In total, we obtained 24 genes of which common SNPs and rare SNPs located in the gene body, including the 5′ UTR and 3’ UTR, were included in our linear mixed model and burden test, respectively (Supplementary Table S1).

### Association Test of Common Variants

**FIGURE 1:**
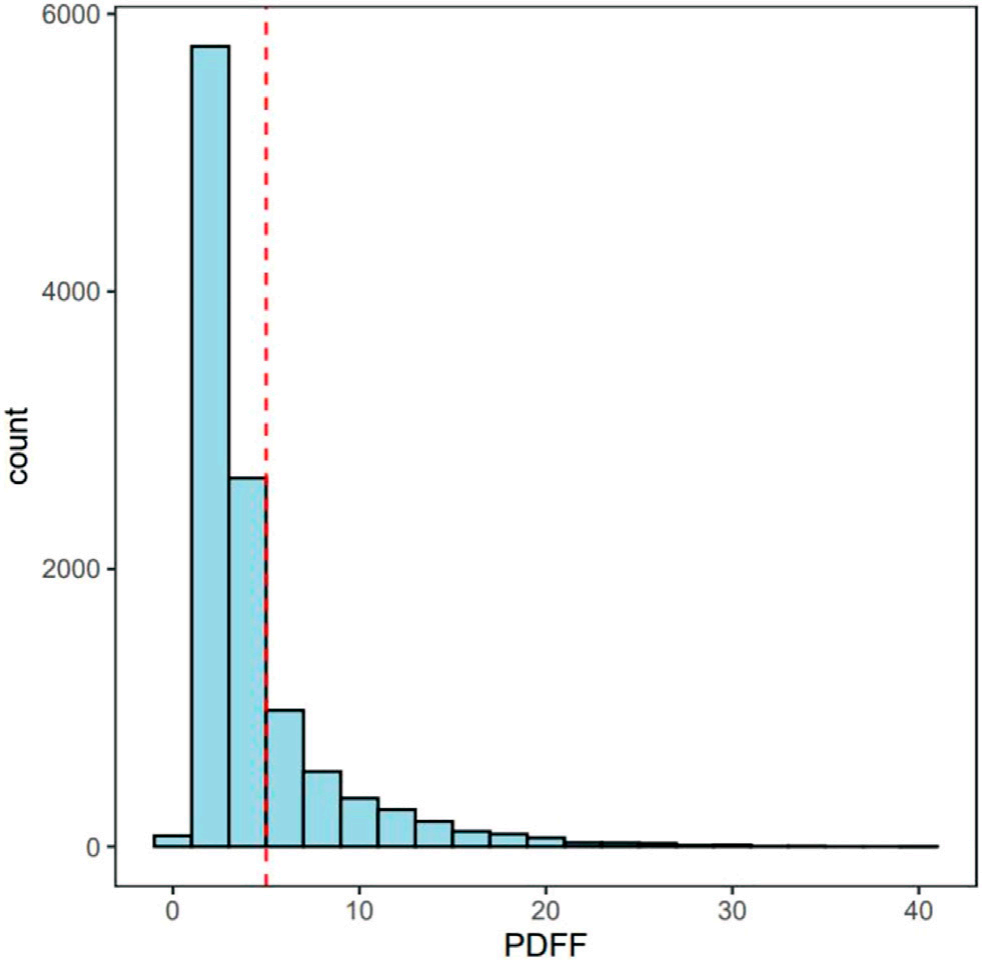
The distribution of PDFF. Red line represents 5% of PDFF. MRI-PDFF < 5% are Non-NAFLD participants and MRI-PDFF ≥ 5% are defined as NAFLD patients.

We conducted the association analysis in a linear mixed model with GCTA (Yang et al., 2011), which takes into account the population structure and kinship. The kinship matrix was calculated using common genetic variants (MAF >0.01%) that passed quality control in all 106 batches and existed in both genotyping arrays. We excluded the participants with excessive alcohol consumption (>40 g per day) or liver damaging drug use to avoid the confounding from nongenetic factors. The PDFF was inverse-normal transformed to a standard normal distribution (Figure 1). We further adjusted sex, age, BMI, and PC1-5 as the covariates in the model. False discovery rate (FDR) was adopted for the multiple tests correction. FDR *p* value < 0.05 was considered as the significant association. Pleotropic effects of the genetic variants were obtained from the NHGRI-EBI GWAS catalog with proxy SNPs (r^2^ > 0.6) or the T2D Knowledge Portal (https://t2d.hugeamp.org/).

For the purpose of picking index SNPs, the common SNPs were clumped by linkage disequilibrium (LD) calculated from 1000 Genomes GBR panel (r^2^ > 0.1, window size of 1 Mb).

### Gene-Based Burden Tests of Rare Variants

In order to study whether the rare genetic variants of glycogen metabolism genes were associated with liver fat fraction, we tested the rare variants (MAF < 0.01) burden in our candidate genes. The rare variants were genotyped by whole exome sequencing using the updated functional equivalence (FE) scheme (called the OQFE scheme). Of the 200 thousand participants who were sequenced, 100,000 variants were observed in the target region covering around 39 million bases. These included 8,086,176 SNPs, 370,958 INDELs, and 1,596,984 multiple allelic variants, with 95.6% of the genetic variants being covered 20 times or more.

Burden tests were evaluated by SAIGE-GENE (Zhou W et al., 2020). Three algorithms were employed, including the tradition burden test, SKAT, which aggregates the individual score test statistics of SNPs in a SNP set and efficiently computes SNP-set level *p*-values (https://www.hsph.harvard.edu/skat/). The Bonferroni corrected *p* value threshold *p* < 0.05 was considered the significant association with PDFF.

### Meta-Analysis of Non-Alcoholic Fatty Liver Disease GWAS

We performed a meta-analysis of the summary statistics of SNPs in glycogen metabolism genes using a fixed-effect inverse variance weighted model. The summary statistics were from four independent GWAS of NAFLD in the populations of European ancestry, included three published NAFLD GWAS (Namjou et al., 2019; Anstee et al., 2020; Liu et al., 2020) and the FinnGen NAFLD GWAS, covering 4,362 NAFLD cases and 602,500 controls. The diagnosis of NAFLD was mostly made using liver biopsy, ultrasound, or computerized tomography. The meta-analysis was conducted using METAL software (Willer et al., 2010).

## Results

### Sample Characteristics

Of the 16,307 participants with PDFF data, 14,056 were of European ancestry. Of these, we excluded 320 participants with hepatic virus infection, 32 participants with liver damaging drug use, and 1,989 participants with excessive alcohol consumption. Finally, 11,715 participants were available for further analysis. Of these, 45.9% were males (Table 1). The average age was 64.3 years (SD = 7.5 years), with an average BMI of 26.5±4.4 kg/m^2^. The average PDFF was 4.35 (SD = 4.04). The average glucose was 4.98 mmol/L (SD = 0.97 mmol/L) and the average triglycerides was 1.66 mmol/L (SD = 0.95 mmol/L). Using the cutoff of 5%, there were 2,466 NAFLD patients, accounting for 21% of the total. The T2D prevalence was 3.5% (414) lower than the population prevalence.

**TABLE 1:** Baseline characteristics of the study population.

**Characteristics**	**—**
N	11,715
Sex (male, %)	5381, 45.9
Age (years)	64.3±7.5
BMI (kg/m^2^)	26.5±4.4
Waist-to-hip ratio	0.87±0.09
PDFF (%)	4.35±4.04
NAFLD (N, %)	2466, 21
T2D (N, %)	414, 3.5
Serum glucose (mmol/L)	4.98±0.97
Triglyceride (mmol/L)	1.66±0.95
Steatosis (N, %)	—
0	8887,75.86
1	2822,24.09
2	6,0.05
3	0,0

Age, BMI, waist-to-hip ratio, PDFF, serum glucose, and triglyceride are given in mean ± SD.

### Common SNP Association Analysis

In total, 11,129 participants with valid genetic data were included in the association tests. We obtained 2,027 common SNPs in the candidate genes from the imputation data (Supplementary Table S2). After LD-based clumping, 30 tagSNPs were selected (Table 2). Nine of these SNPs were significantly associated with PDFF (FDR corrected *p* value < 0.05). These included genes encoding the subunits of enzymes catalyze the reversible conversion between phosphorylated glucose and glycogen, i.e., *GYS2* (rs61928672, beta = −0.04, *p* = 0.006 and rs187630, beta = 0.037, *p* = 0.004), *PYGM* (rs547066, beta = −0.05, *p* = 0.013), and *PYGL* (rs1953873, beta = −0.04, *p* = 0.004), and genes encoding the glucose transporter 1 and 2, i.e., *SLC2A1* (rs2229682, beta = −0.04, *p* = 0.021). Moreover, the glucose kinase (*GCK*) is also associated with PDFF (rs77888691, beta = 0.075, *p* = 0.021). Glucose 6-phosphatase (*G6PC*, *G6PC2/3*) was not associated with PDFF. The genes encoding the regulatory factors, including the β subunits of phosphorylase kinase (*PHKB*) and calmodulin (*CALM2/3*), were also associated with PDFF.

**TABLE 2:** Associations of common SNPs in glycogen metabolism genes with PDFF and NAFLD.

SNP	Chr	POS	EA	OA	EAF	Gene	BETA	SE	P	P_adj	NAFLD_BETA	NAFLD_P
rs2229682	1	43,395,635	C	T	0.79	SLC2A1	0.04	0.014	0.004	**0.021**	−0.025	0.340
rs563702873	2	47,402,220	G	I	0.28	CALM2	0.037	0.013	0.005	**0.021**	0.154	0.871
rs5402	3	170,727,739	A	T	0.12	SLC2A2	0.017	0.018	0.324	0.374	0.135	**2.953E-04**
rs28720688	3	170,729,129	A	G	0.84	SLC2A2	0.005	0.015	0.744	0.744	−0.002	0.950
rs77888691	7	44,231,570	G	T	0.05	GCK	0.075	0.026	0.004	**0.021**	−0.086	0.100
rs10904517	10	5,541,183	T	C	0.34	CALML5	0.018	0.012	0.145	0.218	−0.013	0.571
rs1142825	10	5,567,366	G	A	0.76	CALML3	0.013	0.013	0.311	0.374	−0.010	0.692
rs547066	11	64,523,494	C	A	0.92	PYGM	0.051	0.021	0.013	**0.030**	−0.091	**0.029**
rs61928672	12	21,700,544	G	A	0.85	GYS2	0.044	0.016	0.006	**0.023**	0.027	0.440
rs187630	12	21,746,567	T	C	0.29	GYS2	0.037	0.013	0.004	**0.021**	−0.001	0.941
rs1953873	14	51,392,833	T	C	0.81	PYGL	0.041	0.014	0.004	**0.021**	−0.010	0.772
rs35301423	15	68,487,271	T	I	0.54	CALML4	0.014	0.012	0.238	0.325	−0.024	0.601
rs187431816	16	47,555,554	G	A	0.98	PHKB	0.121	0.047	0.01	**0.025**	NA	NA
rs2593595	17	41,056,245	G	A	0.19	G6PC	0.03	0.015	0.042	0.084	0.004	0.893
rs112003011	17	41,056,283	T	C	0.99	G6PC	0.027	0.048	0.581	0.601	0.244	0.056
rs2229611	17	41,063,466	T	C	0.21	G6PC	0.024	0.014	0.084	0.140	0.013	0.591
rs228758	17	42,148,205	C	T	0.45	G6PC3	0.022	0.011	0.054	0.101	0.031	0.240
rs79646099	19	14,209,826	T	C	0.06	PRKACA	0.016	0.024	0.504	0.560	−0.070	0.201
rs116889014	19	14,225,580	T	G	0.97	PRKACA	0.057	0.032	0.077	0.136	−0.102	0.340
rs56765950	19	47,108,183	C	T	0.36	CALM3	0.019	0.012	0.119	0.188	0.021	0.449
rs10405893	19	47,113,138	G	A	0.89	CALM3	0.043	0.018	0.019	**0.041**	0.005	0.891
rs62125989	19	49,475,876	C	T	0.69	GYS1	0.013	0.012	0.302	0.374	−0.004	0.902
rs7409311	19	49,492,822	G	A	0.93	GYS1	0.022	0.022	0.316	0.374	−0.057	0.209
rs140496340	19	49,495,744	C	I	0.44	GYS1	0.006	0.012	0.577	0.601	NA	NA

EA, effect allele; OA, other allele; EAF, effect allele frequency; BETA, SE, and P, the association summary statistics with PDFF; P_adj, the FDR, corrected P; NA, not available.

The FDR, corrected P or NAFLD *p* values less than 0.05 were highlighted in bold.

**FIGURE 2:**
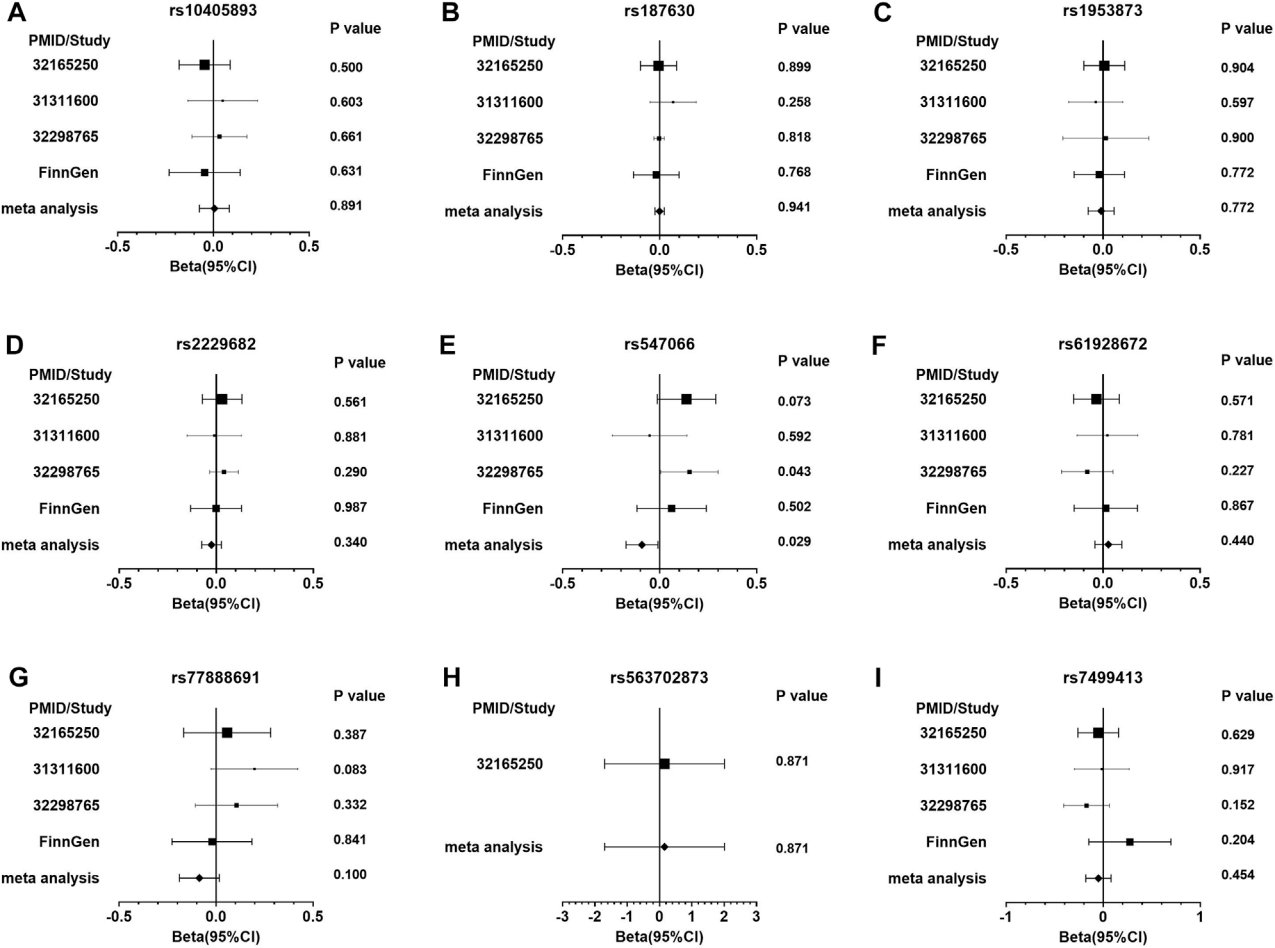
The forest plots of the meta-analysis of NAFLD in glycogen SNPs associated with PDFF. Figure **(A)** presents rs10405893; **(B)** rs187630; **(C)** rs1953873; **(D)** rs2229682; **(E)** rs547066; **(F)** rs61928672; **(G)** rs77888691; **(H)** rs563702873; and **(I)** rs7499413.

The PDFF-associated SNPs were further tested for the association with NAFLD through the meta-analysis of independent studies (Table 2 and Figure 2). Of the nine PDFF-associated SNPs in our study, rs187431816 in PHKB gene was not available in the meta-analyzed result. The proxy SNP rs7499413 (r^2^ = 0.32, D’ = 1.0 with rs187431816) was not associated with NAFLD (*p* = 0.45) in the meta-analysis. Of the remaining eight SNPs, only rs547066 in PYGM gene was associated with NAFLD (*p* = 0.029, Figure 2E). Other SNPs were not significantly associated with NAFLD (*p* > 0.05).

### Pleotropic Effects of the Associated Genes

The glycogen SNPs associated with PDFF were reported to be associated with the serum level of alkaline phosphatase (*PHKB*) and gamma-glutamyl transferase (*GYS2*). Others were associated with glycemic or insulinemic traits, including fasting glucose, glycated hemoglobin, fasting insulin, or type 2 diabetes. *PYGM* and *GCK* were also associated with adiposity traits, e.g., birth weight and BMI (Table 3). In particular, *GCK* and *GYS2* were associated with T2D and fasting insulin level, respectively. It is worth noting that these associations were proxied by SNPs with weak LD with the glycogen SNPs. Hence, we sought to replicate the association with T2D and fasting insulin level using the meta-analyzed results from cohorts that are available in T2D Knowledge Portal. The GYS2 SNP rs187630 was associated with fasting insulin (*p* = 1.16 × 10^−5^), while GCK SNP rs77888691 was not associated with T2D (Supplementary Table S3).

**TABLE 3:** Pleotropic effects of PDFF associated SNPs on liver function or metabolic traits.

SNP	Gene	GWAS_Trait	Proxy_SNP	R^2^	D′	P
rs187431816	PHKB	ALP	rs73536729	0.27	1.00	8.00E-12
rs187431816	PHKB	ALP	rs111617804	0.32	1.00	9.00E-09
rs187630	GYS2	GGT	rs59857578	0.34	0.69	5.00E-20
rs187630	GYS2	Fasting insulin	rs6487237	0.35	0.73	5.00E-09
rs2229682	SLC2A1	Fasting glucose	rs841572	0.15	0.65	5.00E-07
rs547066	PYGM	BMI	rs576076	0.23	0.97	2.00E-12
rs77888691	GCK	Birth weight	rs138715366	0.18	1.00	4.00E-61
rs77888691	GCK	HbA1C	rs2971681	0.16	0.64	1.00E-26
rs77888691	GCK	Fasting glucose	rs10259649	0.13	0.72	2.00E-15
rs77888691	GCK	Type 2 diabetes	rs2908274	0.16	0.64	5.00E-11

ALP, alkaline phosphatase; GGT, gamma-glutamyl transferase; HbA1C, Glycated hemoglobin.

### Rare Variant Burden Test

The rare SNPs of most glycogen metabolism genes were not associated with PDFF after Bonferroni correction (corrected *p* value > 0.05, Table 4). At the nominal significance level, *SCL2A1*, encoding membrane glucose transporter 1, was associated with PDFF (SKAT-O *p* value = 0.005). This association was also supported by the sensitivity analysis made by SKAT and traditional burden test (*p* value = 0.039 and 0.003, respectively).

**TABLE 4:** Burden test of rare SNPs in glycogen metabolism pathway.

Gene	N_marker	SKATO_P	SKAT_P	Burden_P	Burden_BETA	Burden_SE
CALM1	10	1.000	0.973	1.000	2.95E-05	Inf
CALM2	16	0.880	0.724	1.000	6.38E-05	Inf
CALM3	13	1.000	1.000	0.890	−0.002	0.016
CALML3	2	0.757	0.637	0.612	−0.014	0.027
CALML4	8	0.545	0.365	0.520	−0.008	0.012
CALML5	7	0.108	0.073	0.536	−0.010	0.016
CALML6	40	0.073	**0.042**	0.122	0.015	0.010
G6PC	19	0.673	0.787	0.453	0.011	0.015
G6PC2	19	0.052	**0.028**	0.785	−0.002	0.009
G6PC3	6	0.170	0.523	0.110	−0.029	0.018
GCK	12	1.000	0.889	0.927	−0.001	0.011
GYS1	63	0.510	0.318	0.662	0.004	0.008
GYS2	32	0.300	0.178	0.366	0.011	0.012
PHKB	78	0.172	0.601	0.095	0.009	0.006
PHKG1	11	0.346	0.219	0.787	0.004	0.015
PHKG2	30	0.646	0.997	0.439	−0.008	0.010
PRKACA	15	0.672	0.834	0.456	−0.009	0.012
PRKACB	20	0.626	0.920	0.409	−0.009	0.011
PYGB	91	0.844	0.620	0.854	−0.001	0.007
PYGL	54	0.771	0.547	0.632	0.003	0.006
PYGM	80	0.906	0.727	0.901	0.001	0.005
SLC2A1	36	**0.005**	**0.039**	**0.003**	0.032	0.011
SLC2A2	23	0.503	0.325	0.605	0.005	0.009

*p* values less than 0.05 are highlighted in bold. N_marker, number of rare SNPs, included in the test; SKATO_P, the *p* value of SKAT-O, algorithm; SKAT_P, the *p* value of SKAT, algorithm; Burden_P, the *p* value of the traditional burden test. *p* values less than 0.05 were highlighted in bold

## Discussion

In this study, we provided the genetic evidence that liver glycogen storage could bias the liver fat assessment made by imaging-based methods, i.e., PDFF. We discovered that glycogen metabolism genes were associated with liver fat content as measured by PDFF. We also demonstrated that these genes, except for *PYGM*, were not associated with NAFLD. *PYGM* is a glycogen phosphorylase primarily expressed in skeletal muscle, while *PYGL* which was associated with PDFF, is the liver form of glycogen phosphorylase. Hence, our study showed that genetic variants in genes functional relevant to liver glycogen storage could bias the imaging-based liver fat quantification, while not actually associated with the increased risk of excessive live fat deposition.

Glycogen metabolism in the liver involved a pair of reversible reactions which is catalyzed by distinct enzymes. Glycogen synthase 1 and 2 (encoded by *GYS1* and *GYS2*) are responsible for adding glucose to the terminal of the glycogen molecule, while glycogen phosphorylase L and M (*PYGL* and *PYGM*) can cleave a glucose molecule from glycogen. *PYGL* is primarily expressed in the liver. The regulatory proteins of this reversible reaction also play a role in the amount of glycogen stored in the liver. Phosphorylase kinase (*PHK*) and calmodulin could activate glycogen phosphorylases, while protein kinase A is responsible for the activation of *PHK* and deactivation of glycogen synthases. Moreover, the intracellular availability of glucose also affects the glycogen storage in liver. This is usually managed by glucose transporters (*SLC2A1* and *SLC2A2*), glucose kinase (*GCK*), and Glucose-6-phosphatase. The latter two catalyze the reversible reaction that converts between d-glucose and d-glucose 6-phosphate.

Insulin resistance is frequently seen in patients of NAFLD (Watt et al., 2019). Our results showed that *GYS2* genetic variants were associated with both PDFF and fasting insulin. In fact, it has been demonstrated that the genetic disruption of Gys2 in mice induced hepatic glycogen depletion and liver-specific insulin resistance (Irimia et al., 2017). Therefore, the alteration in hepatic glycogen content related to *GYS2* genetic variants may induce the departure of PDFF measurement from the true liver fat content. In addition, the remaining PDFF-associated SNPs were not associated with fasting insulin. This is consistent with our major finding that these genetic variants were not associated with NAFLD.

Glycogen storage in the body is bonded to a large amount of water with a mass ratio of 1:3. MRI radio frequency pulse can form the magnetic coupling between the protons of a glycogen and the nearby water molecules (van Zijl et al., 2007). This magnetic coupling weakens the water signal, which in turn results in exaggerated liver fat measurements. Currently, ^13^C magnetic resonance spectroscopy is a popular glycogen measurement, but a special equipment usually not available in the clinic is required (Gruetter et al., 1994). On MRI, glycogen imaging with chemical exchange saturation transfer imaging (glycoCEST) and nuclear Overhauser enhancement (glycoNOE) can be used to map liver glycogen content indirectly (van Zijl et al., 2007; Zhou Y et al., 2020). However, the difficulty lies in how to decompose the signals of glycogen molecules from the total water signal, since the chemical exchange between the hydroxyl protons of glycogen seems to be inevitable. From a genetics point of view, we demonstrated that the genetic variants of glycogen metabolism genes could bring bias to PDFF, which highlighted the relevance of correcting PDFF for liver glycogen content without the decomposition of glycogen signals from the total.

Our study has limitations. First of all, the participants included in our study and the meta-analysis of NAFLD were mostly of European ancestry. Whether our conclusion can be applied to non-European cohorts remained to be further elucidated. Second, it is possible that the negative associations in our results are attributed to the limited statistical power in NAFLD meta-analysis. Our conclusion should be tested in studies with a larger sample size.

## Conclusion

The common genetic variants in genes related to liver glycogenesis and glycogenolysis were associated with liver PDFF, while they were not associated with the risk of NAFLD. This implicated a bias effect caused by liver glycogen when quantifying the liver fat using MRI. A MRI-based liver fat quantification method simultaneously accounting for liver glycogen storage could lead to a more accurate map of liver fat content.

## Data Availability

Publicly available datasets were analyzed in this study. The UK Biobank data are available through application at the UK Biobank (https://www.ukbiobank.ac.uk/). GWAS summary statistics for NAFLD meta-analysis were obtained through the GWAS catalog (Namjou et al., 2019 and Anstee et al., 2020), personal communication (Liu et al., 2020), and the FinnGen Project (www.finngen.fi).
